# Factors Affecting Pregnancy Rate Following Fallopian Tube Recanalization in Women with Proximal Fallopian Tube Obstruction

**DOI:** 10.3390/jcm7050110

**Published:** 2018-05-10

**Authors:** Mamoon H. Al-Omari, Nael Obeidat, Mwafiq Elheis, Ruba A. Khasawneh, Maha M. Gharaibeh

**Affiliations:** 1 Department of Radiology, Jordan University of Science and Technology, King Abdullah University Hospital, Irbid 22110, Jordan; elheis@hotmail.com; (M.E.); rakhasawneh2@just.edu.jo (R.A.K.); mgharaibeh@just.edu.jo (M.M.G.); 2 Department of Obstetrics and Gynecology, Jordan University of Science and Technology, King Abdullah University Hospital, Irbid 22110, Jordan; naobeidat@yahoo.com

**Keywords:** infertility, fallopian tube, obstruction, recanalization, fluoroscopy, pregnancy rate

## Abstract

Fallopian tube obstruction is a major cause of female infertility. We aimed to evaluate the factors potentially affecting pregnancy rate following fallopian tube recanalization (FTR) in infertile women with proximal fallopian tube obstruction. Data was retrospectively collected for 61 women (25, primary infertility; 36, secondary infertility) who underwent FTR at our institution. Bivariable and multivariable analyses of clinical pregnancy rates in relation to the following factors were performed: primary vs. secondary infertility, duration of infertility, age at the time of FTR, unilateral vs. bilateral obstruction, and previous pelvic interventions. All women who underwent fluoroscopically guided transcervical FTR of one or both proximally obstructed tubes were successfully recanalized (technical success rate, 100%). Within a year after FTR, 41% of women had conceived. None of the studied variables was significantly associated with pregnancy rate on bivariable analysis. Nevertheless, on multivariable analysis, the type and duration of infertility were significantly associated with pregnancy among women aged <35 years at the time of FTR. Our findings regarding pregnancy rates following FTR reflect the diversity of the patient population and suggest the presence of multiple contributing factors. Younger women with secondary infertility for <5 years are highly likely to achieve conception following FTR.

## 1. Introduction

Tubal disease is the cause of subfertility in approximately 30% of women, with 10–25% of such cases due to proximal fallopian tube obstruction (FTO) [1]. Proximal FTO is often caused either by tubal spasm or intrinsic luminal filling defects, such as blood products, debris, minor adhesion, or sinus isthmica nodosa [2]. Such obstruction is easy to treat and amenable for fallopian tube recanalization (FTR) [3]. In contrast, distal FTO is usually caused by pelvic inflammatory disease and frequently associated with extensive peritubal adhesion and hydrosalpinx [3,4]. Distal FTO is not suitable for FTR and is difficult to treat even by other means [4,5].

Proximal FTO is related to the anatomy of the proximal interstitial and isthmic portions of the fallopian tube which are closest to the uterus and have a luminal diameter of approximately 1 mm [6]. The course of this portion is variable, being straight or slightly curved in some women and tortuous in others. This unique anatomy makes these tubal segments prone to spasm, secretion accumulation, mucus plugging, and scarring from inflammation, leading to obstruction and infertility [7].

Proximal FTO is believed to have both a physiological and an anatomical basis. Specifically, the thick muscular wall and reduced proportion of ciliated cells in the epithelium of the proximal tube predispose this tubal segment to spasm [7]. Furthermore, increased tubal secretions at the uterotubal junction and the isthmus during the follicular phase of the cycle can result in stasis of the tubal luminal contents and functional obstruction of the proximal tube. While this accumulation of secretions is typically resolved during the luteal phase of the menstrual cycle, failure to do so may result in prolonged stasis of uterine material, often followed by partial or even complete anatomical obstruction [6].

Selective fallopian tube catheterization with fluoroscopic guidance and use of a coaxial system of guidewires and catheters has been employed since the late 1980s to improve visualization of tubal anatomy and treat proximal FTO [8].

The ability to achieve conception following FTR is believed to be affected by several factors. Specifically, the woman’s age, number of recanalized tubes, type of infertility (primary or secondary), duration of infertility, and history of previous interventions including induced abortion, uterine curettage, and intrauterine devices may all influence pregnancy rate following FTR for infertility related to proximal FTO. The purpose of the present study was to identify factors potentially affecting clinical pregnancy rate after transcervical FTR for proximal FTO.

## 2. Experimental Section

This study was approved by our local ethics review board. Informed consent was obtained from all women included in the study. A total of 61 women with an average age of 34 years (range, 20–45 years) underwent fluoroscopically guided transcervical FTR between February 2007 and June 2011. This is a continuation of an ongoing work on FTR. Our first report for the same patient population has been already published [9]. The previous report was focused on patient preparation, techniques, equipment, tools, compilations, and outcome of FTR. The current report is focused on factors potentially affecting clinical outcome following FTR.

Proximal FTO was confirmed on hysterosalpingogram performed just prior to FTR, thereby reducing the risk of a false positive finding of proximal FTO related to tubal spasm or inadequate tubal filling. All FTR procedures were performed in the angiography suite (Integris 3000; Philips Medical Systems, Eindhoven, The Netherlands), under strict aseptic conditions, using a dedicated FTR kit (FluoroSet; Cook Medical Inc., Bloomington, IN, USA). Initially, a speculum was inserted into the vagina and the cervix was cannulated with a balloon catheter. The cervical balloon was inflated with 2 mL of diluted contrast agent to prevent slippage of the catheter and contrast leak into the vagina. Hysterosalpingography was performed to confirm tubal blockage, and the blocked tube was then cannulated using an angled-tip 5-Fr multipurpose catheter. Selective salpingography was performed to further confirm tubal obstruction. A hydrophilic guide wire (0.035-inch Roadrunner^®^ PC; Cook Medical Inc.) was then inserted into the blocked tube all the way until the wire could move freely within the peritoneal cavity. Finally, tubal patency and peritoneal spillage of contrast agent were confirmed on selective salpingogram. A detailed description of the procedure, including the device used, initial hysterosalpingography, selective salpingography, and FTR, is available elsewhere [9].

All women were admitted to the day care unit for preprocedural preparation and postprocedural observation. Analgesics were given to all women. After FTR, the women were advised to avoid sexual intercourse for a few days, particularly if bleeding or vaginal spotting occurred. Regular follow-up was performed in the outpatient clinics, both via visits and by telephone.

The following inclusion criteria were applied: (i) infertility for at least 1 year, and (ii) prior hysterosalpingogram or laparoscopic examination demonstrating unilateral or bilateral fallopian tube occlusion. Women whose partners had male-related infertility factors and those with endometriosis, previous salpingectomy, ectopic pregnancy pelvic inflammatory disease, or evidence of distal tubal blockage were excluded from the study.

The medical records and imaging data of all women treated at our institution during the study period and who met the inclusion criteria were reviewed for extracting demographic and clinical information. The pregnancy rate within the first 12 months following FTR was recorded. The association between conception rate and the following factors was assessed: primary vs. secondary infertility, duration of infertility, age at the time of FTR, unilateral vs. bilateral obstruction, and previous pelvic intervention.

### Statistical Analysis

Data was analyzed using Statistical Package for the Social Sciences (SPSS) software version 24 (SPSS Inc., Chicago, IL, USA). Pearson chi-square test was used to compare the post-FTR pregnancy rates according to relevant characteristics. Multivariable analysis using binary logistic regression was used to determine the predictors of successful conception after FTR for women with age at FTR <35 years and ≥35 years, separately. The adjusted odds ratios and their confidence intervals were calculated and reported. A *p*-value < 0.05 was considered statistically significant.

## 3. Results

Among the 61 women who underwent fluoroscopically guided transcervical FTR during the study period, approximately two thirds had bilaterally blocked tubes and one third had unilateral proximal FTO on selective salpingography. The mean duration of infertility was 4 years (range, 1–14 years), with primary infertility in 25 women (41%) and secondary infertility in 36 women (59%). All women had undergone previous hysterosalpingography, while 31 had received diagnostic laparoscopy. Five women (13%) had prior ectopic pregnancies (right side, 3 women; left side, 2 women) treated with salpingectomy. Twenty-six women had a history of previous pelvic interventions including intrauterine contraceptive device (IUCD) implantation, dilation, and curettage.

All women were successfully recanalized, with an immediate postprocedural patency rate of 100%. On follow-up for up to 1 year after FTR, 41% of the women had conceived, with all pregnancies being intrauterine. Post-FTR pregnancy rates according to relevant characteristics are shown in Table 1. The pregnancy rates for women with primary and secondary infertility were 32% and 47.2%, respectively, with no statistically significant difference (*p* = 0.234). Although not statistically significant (*p* = 0.076), the pregnancy rate was higher for women with a duration of infertility <5 years than for women with a duration of infertility ≥5. Overall, none of the studied variables was significantly associated with pregnancy rate on bivariable analysis (Table 1).

When stratified according to age at time of FTR, the type and duration of infertility were significantly associated with pregnancy among women aged <35 years at the time of FTR. However, none of the studied variables were significantly associated with pregnancy among women aged ≥35 years at the time of FTR (Table 2).

On multivariable analysis (Table 3), only the type and duration of infertility were significantly associated with pregnancy among women aged <35 years at the time of FTR. In this patient population, secondary infertility was associated with a 15-fold increase in the odds of conception compared with the odds noted in women with primary infertility, whereas infertility with a duration of <5 years was associated with a 21-fold increase compared with the odds noted in women with longer-duration infertility.

## 4. Discussion

Our study showed a significant interaction effect between women’s age at the time of FTR with the type and duration of infertility on pregnancy rate. Findings presented in the current study may partially explain the conflicting evidence regarding clinical success rate following this procedure.

After the introduction of FTR as a treatment for proximal FTO [8], several studies have confirmed the safety and efficacy of this procedure [8,9,10]. However, there is substantial variation in the reported technical success rates and pregnancy rates following FTR. Reported variations in technical success rates may be attributed to operator experience, tools, and equipment used [9].

In their study of 153 women with tubal infertility, Tanaka and Tajima [11] reported a pregnancy rate of 28.9%. Al-Omari et al. [9] reported a pregnancy rate of 41% within the first year following FTR, while Seyam, Hassan et al. [12] found a cumulative pregnancy rate of about 26% following any of two different FTR procedures. Higher pregnancy rates (51%) were reported by Mallarini and Saba [13], whereas lower rates (12.8%) were reported by Lang and Dunaway [14]. These conflicting results are likely related to several factors.

Those variations in pregnancy rates following FTR has not been thoroughly investigated to date. Jacqueline et al. [15] did not find any prognostic factors affecting cannulation rate and pregnancy rate in a cohort of fifty women after successful hysteroscopic proximal tubal cannulation in relation to age, type of infertility, duration of infertility, and history of pelvic inflammatory disease. Spyros Papaioannou et al. [16] showed that conception rates were significantly higher in women younger than 35 years, but there was no significant difference identified according to whether bilateral or unilateral tubal blockage was present at time of recanalization.

In the present study, we assessed the pregnancy rate following FTR in relation to different parameters expected to affect conception, including age at the time of FTR, type of infertility (primary vs. secondary), number of successfully recanalized tubes (unilateral vs. bilateral), duration of infertility, and history of pelvic surgery or IUCD insertion. We found significant variation in patient epidemiology and pregnancy rates, which may partially explain the conflicting evidence reported to date regarding the clinical outcomes of FTR. Specifically, we found no statistically significant association between pregnancy rates and examined variables on bivariable analysis.

We found that pregnancy rates tended to be higher for women with secondary infertility than for those with primary infertility (47.2% vs. 32%). Women who had previous successful conception are typically expected to have a higher chance of becoming pregnant after treatment of the underlying cause (in this case, proximal FTO). Since FTO is reportedly common in secondary infertility, some women with primary infertility and proximal FTO may have other idiopathic cofactors that could affect their fertility status [17].

Furthermore, we found that pregnancy rates tended to be higher for women with duration of infertility <5 years than for those with duration of infertility ≥5 years (48.8% vs. 25%), which may indicate that long-term tubal obstruction by inflammatory reaction, which is the most common pathological finding in women with proximal FTO [18], may affect tubal internal structures including the cilia. Therefore, long-term tubal obstruction makes pregnancy more difficult even in women with normal-looking tubes after recanalization, potentially increasing the risk of reocclusion after recanalization. Thus, we recommend prompt treatment of proximal FTO.

Upon stratifying the study population according to age at the time of FTR, we found that pregnancy rate was higher in women younger than 35 years than in older women (50% vs. 31%). Moreover, younger women showed a significant association of post-FTR pregnancy rate with type and duration of infertility, whereas no significant association was noted among older women. The inverse association between pregnancy rate and advancing maternal age is well known, with many contributing factors [16,19].

On stratification according to the number of recanalized tubes, we found that successful recanalization led to a higher pregnancy rate in women with bilateral obstruction than in those with unilateral obstruction (46.2% vs. 31.8%), suggesting that women with unilateral obstruction may have other factors contributing to their infertility. Indeed, Hayashi, Hoshimoto, and Ohkura [20] showed that unilateral obstruction may affect the physiology of the contralateral patent tube. Thus, we strongly recommend FTR in women with unilateral obstruction, as it increases the chance of pregnancy, but other possible causes for infertility should be thoroughly investigated.

A history of pelvic surgery or IUCD implantation is reported to affect tubal patency and thus pregnancy rate [21]. Additionally, previous induced abortion, uterine curettage, pelvic inflammatory disease, and intrauterine device implantation have been related to partial FTO infertility [18,22]. While we also found that patients with a previous history of such intervention had a higher pregnancy rate following FTR (46.2% vs. 37.1% in women without such history), the association was not statistically significant on either bivariable or multivariable analysis.

On bivariate analysis, pregnancy rates varied with one factor or another, but these variations were not statistically significant, mostly due to the small size of the subgroups or perhaps the presence of confounding factors within the same patient. Therefore, we analyzed the data in a multivariable manner, and found interesting results. Specifically, age at the time of FTR was the most important factor affecting post-FTR conception rates. Advanced maternal age is a well-known risk factor for female infertility, as fertility in women was reported to peak at 25 years and subsequently decline, with a dramatic drop after 35 years [19]. Importantly, we found that both the type and duration of infertility were significantly associated with pregnancy among women aged <35 years at the time of FTR (secondary infertility: pregnancy rate, 73.3%, with odds ratio 15.1 vs. primary infertility; infertility duration <5 years: pregnancy rate, 66.7%, with odds ratio 21.0 vs. infertility duration ≥5 years). This finding indicates that women aged <35 years at the time of FTR and having secondary infertility with a duration of <5 years have a very high chance to become pregnant after FTR.

This study has two major limitations. First, the sample size was small, particularly on stratification into subgroups; therefore, there was no power analysis to determine whether each subgroup was adequately powered to detect meaningful differences. Second, no control group was included. Other limitations include the observational, retrospective design. However, the results were encouraging and warrant further confirmation via randomized control trials with large sample size.

## 5. Conclusions

Despite its limitations, the present study brings evidence that pregnancy rates following FTR vary significantly, reflecting the diversity of the patient populations and the presence of multiple contributing factors. Age at the time of FTR seems particularly significant. Our findings suggest that younger patients with secondary infertility lasting for less than 5 years have an extremely high chance of becoming pregnant following FTR.

## Figures and Tables

**Table 1:** Post-FTR pregnancy rates according to relevant characteristics.

Characteristic	Pregnancy					
	No		Yes		Total *n*	*p*-Value *
	*n*	%	*n*	%		
Type of infertility						0.234
Primary	17	68.0	8	32.0	25	
Secondary	19	52.8	17	47.2	36	
Duration of infertility						0.076
<5 years	21	51.2	20	48.8	41	
≥5 years	15	75.0	5	25.0	20	
Obstruction						0.274
Unilateral	15	68.2	7	31.8	22	
Bilateral	21	53.8	18	46.2	39	
Age at FTR						0.133
<35 years	16	50.0	16	50.0	32	
≥35 years	20	69.0	9	31.0	29	
Abdominal operations						0.479
No	22	62.9	13	37.1	35	
Yes	14	53.8	12	46.2	26	

* Pearson chi-square test; FTR, fallopian tube recanalization.

**Table 2:** Post-FTR pregnancy rates according to age at the time of FTR.

Characteristic	Age at FTR									
	<35 Years					≥35 Years				
	Pregnancy					Pregnancy				
	No		Yes		*p*-Value	Yes		No		*p*-Value
Type of infertility	*n*	%	*n*	%	0.013 *	*n*	%	*n*	%	0.642
Primary	12	70.6	5	29.4		5	62.5	3	37.5	
Secondary	4	26.7	11	73.3		15	71.4	6	28.6	
Duration					0.009 *					0.858
<5 years	7	33.3	14	66.7		14	70.0	6	30.0	
≥5 years	9	81.8	2	18.2		6	66.7	3	33.3	
Obstruction					1.000					0.160
Unilateral	5	50.0	5	50.0		10	83.3	2	16.7	
Bilateral	11	50.0	11	50.0		10	58.8	7	41.2	
Operations					0.102					0.732
No	14	58.3	10	41.7		8	72.7	3	27.3	
Yes	2	25.0	6	75.0		12	66.7	6	33.3	

* Statistically significant using Pearson chi-square test; FTR, fallopian tube recanalization.

**Table 3:** Predictors of successful conception after FTR, according to age at the time of FTR.

Predictor	Age at FTR			
	<35 Years		≥35 Years	
	OR (95% CI)	*p*-Value	OR (95% CI)	*p*-Value
Type of infertility				
Primary	1.0		1.0	
Secondary	15.1 (1.6, 146.8)	0.019 *	0.6 (0.1, 3.7)	0.599
Duration of infertility				
<5 years	21.0 (1.8, 245.8)	0.015 *	0.8 (0.1, 4.4)	0.759
≥5 years	1.0		1.0	

Data obtained via multivariable logistic regression analysis. * Statistically significant, as indicated by *p* < 0.05. CI, confidence interval; FTR, fallopian tube recanalization; OR, odds ratio.
